# Impact of Medical Cannabis on Recovery from Playing-Related Musculoskeletal Disorders in Musicians: An Observational Cohort Study

**DOI:** 10.3390/healthcare12131335

**Published:** 2024-07-04

**Authors:** Kathryn Cottrell, John Chong

**Affiliations:** Musicians’ Clinics of Canada, Hamilton, ON L9C 7N4, Canada

**Keywords:** medical cannabis, playing-related musculoskeletal disorders, musicians, pain, depression, stress

## Abstract

Introduction: Playing-related musculoskeletal disorders (PRMDs) are musculoskeletal symptoms that interfere with the ability to play at the level a musician is accustomed to. Musicians have an 84% lifetime prevalence of PRMD. Many types of analgesia are inappropriate for this population due to their risks, but cannabidiol (CBD) has been shown to have anti-inflammatory properties and can reduce the perception of pain. Medical cannabis has also been shown to be safer than other analgesia in terms of serious adverse events. This study explores the impact of medical cannabis for PRMD on perceptions of pain and mental health outcomes. Methods: Participants (*n* = 204) completed questionnaires at baseline and six months: the Musculoskeletal Pain Intensity and Interference Questionnaire for Musicians (MPIIQM) and Depression, Anxiety and Stress Scale (DASS-21). Participants self-selected their group: non-cannabis users (*n* = 42), new medical cannabis users (*n* = 61), and long-term medical cannabis users (*n* = 101). Data were analyzed using paired *t*-tests for within-group and ANOVA for between-group differences. Results: At six months, there was no difference (*p* = 0.579) in cannabidiol dose between new (24.87 ± 12.86 mg) and long-term users (21.48 ± 12.50 mg). There was a difference in tetrahydrocannabinol (THC) dose (*p* = 0.003) between new (3.74 ± 4.22 mg) and long-term users (4.41 ± 5.18 mg). At six months, new cannabis users had a significant reduction in pain intensity as measured by The Musculoskeletal Pain Intensity and Interference Questionnaire for Musicians (MPIIQM40) (*p* = 0.002). Non-users (*p* = 0.035), new users (*p* = 0.002), and long-term cannabis users (*p* = 0.009) all had significant reductions in pain interference (MPIIQM50) at six months. At six months, non-cannabis (*p* = 0.022) and long-term cannabis users (*p* = 0.001) had an improvement in DASS-21. The change in pain intensity was the only difference between groups, F(2, 201) = 3.845, *p* = 0.023. This difference was between long-term (0.83 ± 0.79) and new users (−2.61 ± 7.15). No serious adverse events occurred, and a minority experienced tiredness, cough, and dry mouth. Discussion/Conclusions: This practice-based evidence demonstrated that the multidimensional approach to care provided by the Musicians’ Clinics of Canada benefited all groups at six months. Medical cannabis significantly reduced pain intensity in new users of medical cannabis with PRMD, and all groups saw improvements in pain interference. In keeping with prior studies, medical cannabis seems to be effective at reducing perceptions of pain, including for PRMD. CBD/THC dosing was within guideline recommendations, and no patients experienced any serious adverse events. Limitations include multiple factors impacting patients’ decisions to opt in or out of medical cannabis. These include cost, comorbidities, and disease chronicity. In conclusion, medical cannabis reduces pain intensity in new users, and when combined with a multidimensional approach to care, patients with PRMD can see improvements in pain as well as mental wellbeing.

## 1. Introduction 

### 1.1. Background 

Playing-related musculoskeletal disorders (PRMDs) are defined as ‘pain, weakness, numbness, tingling or other symptoms that interfere with the ability to play the instrument at the level you are accustomed to’ [1]. Risk factors for PRMD include previous injuries, music performance anxiety, experiencing high levels of stress, long practice hours, and obesity [2,3]. Musicians have an 84% lifetime prevalence of PRMD [4], and treatment options are limited. This is in part because although PRMD is a physical health condition, it also has an impact on musicians’ mental health, financial stability, and sense of self [1,5]. This is similar to experiences of elite athletes, as for both groups, physiological, anatomical, and psychosocial influences all play a significant role in the prevalence and impact of pain [6]. As a result of the limited treatment options, many patients are being managed with opioids [7,8], putting them at risk of serious adverse side-effects and addiction [9]. In Canada alone, approximately 2000 people die from opioid-related poisonings each year [10]. In terms of non-steroidal anti-inflammatory drugs as a treatment option, these are associated with gastrointestinal side-effects, and can also mask the pain, potentially increasing the risk of musicians playing through the pain and worsening the initial injury [11]. Medications such as gabapentin and pregabalin, often used for pain management, can also cause significant adverse effects with mixed analgesic results [12]. 

Cannabis has been used for medicinal purposes for at least 5000 years [13]. The two most well-studied components of cannabis are tetrahydrocannabinol (THC) and cannabidiol (CBD), both of which act on the endocannabinoid system [14]. However, there are over 400 known chemical entities within cannabis including many that have biologically active metabolites for which research is growing [13]. The endocannabinoid system plays an important role in physiological, behavioral, immunological, and metabolic functions [15], including the regulation of pain sensation [16]. There is minimal research on the use of medical cannabis for pain, but the studies that do exist show some potential benefit [17,18]. A cross-sectional study of cannabis users shows that the top three medical conditions people use it for are pain, anxiety, and depression [19]. CBD specifically has been shown to have sedative, anti-inflammatory, and neuroprotective properties [20], and to positively influence the perception of pain across different conditions [21]. In this paper, when referring to ‘cannabis’ or ‘medical cannabis’, this encompasses all cannabinoids within the plant versus when CBD and THC are referenced specifically. 

Due to occupational hazards, musicians are at high risk for addictions [22], and those with PRMD have often already exhausted many standard treatment approaches. Medical cannabis has been shown to be significantly safer than opioids in terms of serious adverse events [23], with the main potential harms being cognitive impairment, drowsiness, and impaired attention, most of which are self-limiting and transient [21]. Therefore, due to its potentially beneficial effects, alongside its relatively robust safety profile, medical cannabis has been recommended in the treatment and optimization of chronic non-cancer pain, following principles of harm reduction and risk minimization [24]. This recommendation was echoed in a recent clinical practice guideline published by Busse et al. which showed that medical cannabis can result in improvements in self-reported pain intensity, physical functioning, and sleep quality [21]. This guideline recommends offering a trial of non-inhaled medical cannabis if standard care is not sufficient for symptom control. MacCallum et al. demonstrated that when used as an adjunctive therapy to opioids, medical cannabis achieved better pain control with lower doses of opioids, improved pain-related outcomes, and reduced opioid-related harm [24]. There is no published research on the use of cannabis for the treatment of PRMD. Therefore, this study aims to provide some initial findings by gathering more information on the efficacy and safety of medical cannabis for PRMD for musicians and their care providers. 

### 1.2. Objectives 

The objectives of this study are to determine whether medical cannabis reduces pain intensity experienced by patients with PRMD, as well as the amount of interference caused by their pain. A secondary objective is to determine whether medical cannabis makes a difference to patients’ levels of stress, anxiety, and depression, as well as sleep quality, as these are often linked with the perception of pain. These findings will help us determine whether medical cannabis is a viable, effective, and safe option for the management of PRMD. 

## 2. Materials and Methods 

### 2.1. Study Setting and Participants 

This quantitative, observational cohort study was completed at the Musicians’ Clinics of Canada, an Ontario Health Insurance Plan (OHIP)-funded clinic that receives referrals for musicians with a host of different health conditions. Interventions at the clinic include biofeedback such as surface electromyography, motion analysis, heart rate variability, and neurofeedback, as well as lifestyle interventions, psychotherapy, and pharmacological management, which includes medical cannabis, where appropriate [25]. Patients with PRMD are informed about the current evidence for medical cannabis as well as the risks and benefits, before shared decision making occurs between patient and physician to decide whether they want to include medical cannabis in their treatment plan. In Canada, recreational cannabis has been legalized since October 2018, prior to the start of this study [26]. 

Patients over the age of 18 who received treatment in-person for PRMD between January 2019 and January 2020, and gave written consent, were enrolled into this study. Patients with active cancer, previous or current cannabis use disorder, and psychotic disorders were excluded. The diagnosis of PRMD was based on Zaza et al.’s definition published in 1998 [1]: pain, weakness, numbness, tingling, or any other symptoms that interfere with the patients’ ability to play their instrument at the level they are accustomed to, which does not include transient aches or pains [1,27]. Demographic variables collected from study participants included age, sex, adverse childhood experiences [28], and the use of analgesics. Data were collected from their baseline visit, and a subsequent visit approximately six months later. The study population comprised non-cannabis users (no recent cannabis use, and not prescribed medical cannabis as per patient choice), new cannabis users (for whom we have baseline data prior to starting medical cannabis), and long-term cannabis users (who used medical cannabis prior to the start of the study period). Medical cannabis was prescribed in line with the consensus recommendations by Bhaskar et al. on optimal dosing for neuropathic, inflammatory, nociplastic, and mixed pain [29]. Patients who chose to include medical cannabis in their treatment plan were all started on low-dose (usually 5 mg of CBD daily), non-inhaled, oral products from a licensed producer, with a gradual dose increase depending on symptom response and shared decision making. Small doses of THC were added as needed for further symptomatic relief. This study was approved by the Hamilton Integrated Research Ethics Board (ref: 2021-11397-GRA). 

### 2.2. Study Design and Outcome Measures 

The primary outcome measures were based on three validated questionnaires: the Musculoskeletal Pain Intensity and Interference Questionnaire for Musicians (MPIIQM) [30], which is a modification of the Brief Pain Inventory; the Depression, Anxiety and Stress Scale (DASS-21) [31]; and the Pittsburgh Sleep Quality Index (PSQI) [32]. The MPIIQM includes two scores which give an objective measure of PRMD symptoms: one for pain intensity (0–40) and one for pain interference (0–50) [30]. DASS-21 and PSQI were included as it has been demonstrated that musicians who experience anxiety, depression, or poor sleep have more severe PRMD symptoms [2,3]. Changes in scores from baseline to six months were compared to explore the impact of medical cannabis on their symptoms. The secondary outcome measure, self-reported adverse events, were collected at each visit during the investigation period by completion of a written form. 

### 2.3. Statistical Methods 

The sample size was pre-set as it is a convenience sample from the Musicians’ Clinics of Canada. Between-group differences in baseline patient characteristics were compared using X^2^ tests for categorical characteristics and one-way ANOVAs for continuous characteristics. The means and standard deviations for the continuous dependent variables were calculated for the baseline and six-month follow-up. For each individual group (non-users, new users, long-term users), paired-sample *t*-tests were used to determine whether there was a statistically significant mean difference between questionnaire scores before and after the intervention. Multiple one-way ANOVAs were conducted to determine if there was a statistically significant difference in MPIIQM, DASS-21, and PSQI scores between the three groups at the six-month point, to see if medical cannabis made a difference compared to other treatment interventions in the clinic. There were outliers in the ‘new cannabis users’ group for depression and PSQI scores as assessed by a boxplot, but these were accurate numbers and were therefore kept in the dataset. There was a homogeneity of variances, as assessed by Levene’s test of homogeneity of variances.

## 3. Results 

### 3.1. Participants and Descriptive Data 

**Figure A1:**
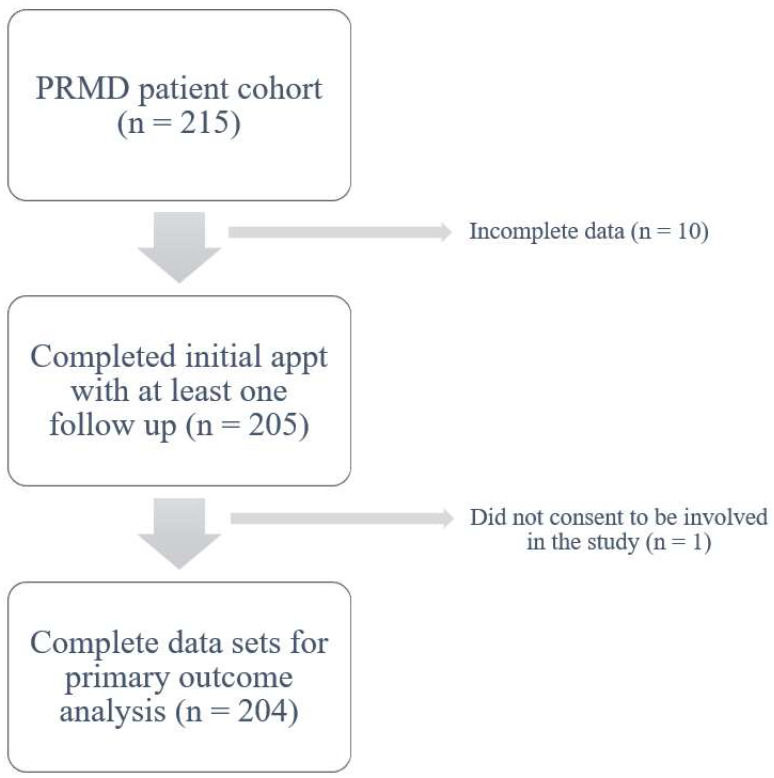
Flow diagram of study participants.

Figure A1 illustrates the patient flow through this study. A total of 215 patients fulfilled eligibility for recruitment into this study. A total of 205 of these completed at least one follow-up visit. One person withdrew their consent for being involved in this study. Complete datasets were available for the remaining 204 patients: non-cannabis users (*n* = 42), new cannabis users (*n* = 61), and long-term cannabis users (*n* = 101). There was no statistically significant difference in baseline characteristics between groups as outlined in Table 1, nor between baseline questionnaire scores, outlined in Table 2. 

### 3.2. Outcome Data: Main Results 

The results of medical cannabis dosing within this study are displayed in Table 3; data are presented as mean ± standard deviation. All medical cannabis used was administered orally as an oil from a licensed producer of the patients’ choosing. At six months, the new cannabis users were using 24.87 ± 12.86 mg of CBD and 2.11 ± 1.45 mg of THC daily. At baseline, the long-term medical cannabis users were using 21.48 ± 12.50 mg of CBD and 3.74 ± 4.22 mg of THC. There was no statistically significant difference in CBD or THC dosage from baseline to six months in the long-term cannabis users group as assessed by paired *t*-tests, with six-month use at 23.39 ± 15.60 mg of CBD (*p* = 0.074) and 4.41 ± 5.18 mg of THC (*p* = 0.080) each day. There was no statistically significant difference in CBD dose between the new users and long-term users at six months (*p* = 0.579) as assessed by independent *t*-tests. However, long-term users did use a significantly higher dose of THC compared to new users at six months (*p* = 0.003). 

Results of MPIIQM, DASS-21, and PSQI scores are displayed in Table 4. The data are presented as mean ± standard deviation. At six months, within the non-cannabis user group, there was a statistically significant improvement in MPIIQM50 (*p* = 0.035), stress (*p* = 0.043), anxiety (*p* = 0.027), and overall DASS-21 score (*p* = 0.022). At six months, within the new cannabis user group, there was a statistically significant improvement in MPIIQM40 (*p* = 0.006), MPIIQM50 (*p* = 0.002), and overall MPIIQM score (*p* = 0.001). At six months, within long-term cannabis users, there was a statistically significant improvement in MPIIQM50 (*p* = 0.009), stress (*p* = 0.010), anxiety (*p* = 0.004), depression (*p* = 0.002), and overall DASS-21 score (*p* = 0.001). 

Results for changes in MPIIQM, DASS-21, and PSQI scores from baseline to six months are displayed in Table 5. Data are presented as mean ± standard deviation. The change in pain intensity score, as assessed using the MPIIQM40 tool, was the only statistically significant difference between the different groups, F(2, 201) = 3.845, *p* = 0.023. MPIIQM40 differences increased from long-term cannabis users (0.83 ± 0.79) to non-cannabis users (−0.48 ± 7.65), to new-cannabis users (−2.61 ± 7.15). Tukey–Kramer post hoc analysis revealed that the statistically significant difference was between the new cannabis users compared to the long-term cannabis users (*p* = 0.017). The difference in group means for the change in score at six months for overall MPIIQM, DASS-21, and PSQI scores, as well as MPIIQM50, anxiety, depression, and stress individually, was not statistically significant (*p* > 0.05). 

### 3.3. Outcome Data: Secondary Analyses

There were no self-reported or observed serious adverse events in any patients using medical cannabis during the study period. Unwanted effects were experienced by a small number of patients including tiredness, overeating, cough, dry mouth, and short-term subjective cognitive impairment. Data for adverse events were recorded qualitatively on a patient questionnaire and discussed with the physician at each visit.

## 4. Discussion 

All three groups (non-cannabis users, new users, and long-term) had statistically significant improvements in some of the measured domains at six months. For the non-cannabis users, this was in pain interference, stress, anxiety, and overall DASS-21 score, and these findings were echoed in long-term users, although they also experienced a significant improvement in depression from baseline. In the new-cannabis users group, the significant differences were only noted in pain intensity and interference scores with no significant change in mental health measures. These findings suggest that the multidimensional model of care provided by the Musicians’ Clinics of Canada plays a significant role in symptom improvement for patients presenting with PRMD. Although there is very little research into the treatment of PRMD, these findings are in keeping with recommendations from the American College of Physicians [33], the Canadian Guidelines for opioid therapy and chronic non-cancer pain [8], and the International Olympic Committee consensus on pain management in elite athletes [34], who recommend the use of non-pharmacological treatments for pain such as acupuncture, mindfulness-based stress reduction, motor control exercises, progressive relaxation, electromyographic biofeedback, and cognitive behavioral therapy, all of which are on offer for patients attending the clinic. 

The main finding was a statistically significant reduction in intensity of pain as measured by the MPIIQM tool in new cannabis users versus long-term cannabis users at the six-month mark. This is in keeping with findings reported by Busse et al. in their recently published clinical practice guideline which showed an important improvement in pain for patients with chronic pain on medical cannabis [21]. Long-term cannabis users may have already experienced their initial reduction in pain intensity when treatment was initiated, reaching a steady state by the time data were collected for this study. It is also important to note that long-term cannabis users were using more THC than new users at the six-month mark, which may have impacted our results. This may be due to differences in pain severity and chronicity of PRMD between the groups and correlates with previous research which shows a more beneficial role for THC compared to CBD in pain attenuation [35]. Additionally, tolerance to THC may have developed in long-term users, a phenomenon well established in the literature studying frequent cannabis users [36]. The finding of a reduced intensity of pain in new users with medical cannabis is critical to this population, as this reduction in pain may enable them to continue their work and reduce the risk of chronic functional impairment and disability. 

In terms of the physiology behind cannabis’ effect on pain, we must look at the endocannabinoid system, an important neuromodulatory system involved in both synaptic and immune modulation. It has receptors in the central nervous system, peripheral, immune, and hematologic systems [21]. It has been shown that type 2 cannabis receptors (CB2) are upregulated by microglia during neuroinflammation and other insults in an attempt to counteract inflammation [37]. Modulation of the endocannabinoid system, particularly CB2 receptors, is likely one of the mechanisms by which medical cannabis can modulate the response to pain. This has been demonstrated in studies which show that CB2 agonists diminish pain sensitivity [38,39]. This occurs by reducing neuroinflammation through the microglia, the resident immune cells of the brain [37]. The beauty of microglia is that they can proliferate on demand and renew themselves, making them an ideal treatment target [40,41]. 

Results from this study showed that there was no significant difference in mental health between the three different groups. This may have been due to the positive effects experienced by the multiple non-pharmacological interventions on offer to all patients in this study and potential unmeasured underlying differences between the groups at baseline. There was no significant difference between groups for sleep quality with or without cannabis, which is in keeping with prior studies that have shown mixed results for sleep latency, sedation, and the sleep–wake cycle [42]. 

In terms of side-effects, there were no serious adverse events within our population caused by medical cannabis from a licensed producer. However, this study does not look at long-term effects and safety, recreational cannabis, nor at special populations such as children. A common concern with cannabis is the risk of psychosis, but as reviewed by Busse et al. [21], no studies using medical cannabis in adults have identified an association between medical cannabis and early-onset psychosis [43,44]. Our safety profile is in keeping with other research that shows that medical cannabis is well tolerated [45,46]. 

This practice-based evidence is important to enable patients with PRMD to feel better informed to make healthcare decisions. This is particularly important due to the financial burden associated with medical cannabis compared to other analgesia. Socioeconomic factors likely contributed to patient treatment choice as medical cannabis is not covered by benefits or OHIP, and costs approximately 4.50 CAD a day for the recommended dose of 40 mg of CBD in medical cannabis. This is a significant burden, especially for musicians who earn 44% less than the average Canadian worker [47]. In addition, a variability in dosing across patients within this study reflects how medical cannabis is used by patients in the community, with patients increasing and decreasing their dose based on symptoms, travel restrictions, and income. This is particularly true for musicians who often travel internationally with their work, including to countries where cannabis is illegal [48]. It must also be noted that even though this paper looked for statistically significant improvements, there will be considerable variability in how much change in pain, stress, depression, and anxiety and improvement in sleep patients would deem clinically important for them on an individual basis. Further qualitative analysis is required to explore patients’ individual experiences with the use of medical cannabis for their PRMD. 

### Limitations

Our population was relatively heterogeneous, with a significant variation in severity of pain as well as mental health status, as demonstrated by the number of outliers. Our group sizes were also considerably different, with more patients choosing to try medical cannabis, and a relatively small control group. There is a high risk of bias due to the lack of randomization and self-selection of the group by patients. As patients self-selected whether to use medical cannabis or not, we must consider factors such as the impact of their PRMD on quality of life and the number of prior pharmacological treatments tried, to determine what factors made people more likely to try medical cannabis as this may have affected our results. There is a risk of bias due to self-reported outcomes; however, these were chosen purposefully as subjective outcomes are of upmost importance with regard to pain symptoms. Medical cannabis administration also varied significantly in terms of formulation and dose, which may have had pharmacokinetic and pharmacodynamic implications. Further larger studies, with placebo control, are required to determine the optimal dosing of medical cannabis; to further explore its impacts on pain, sleep, and mental health conditions; and to assess long-term adverse effects. It is also important to establish the numbers needed to treat and numbers needed to harm for medical cannabis, so a true comparison with other pharmacological options can be made. The limited number of randomized controlled trials looking at medical cannabis, and the significant heterogeneity between study populations and treatment characteristics, means more evidence is required. 

## 5. Conclusions 

In conclusion, within our study population over a six-month period, medical cannabis proved to be a safe and potentially beneficial treatment option for musicians with PRMD, with those using medical cannabis for the first time seeing a statistically significant reduction in pain intensity. All patient groups experienced an improvement in some domains of pain experience or mental wellbeing, likely due to the multidimensional model of care. Many patient concerns about medical cannabis include adverse drug effects, addiction, tolerance, losing control, or unusual behavior [21], but hopefully this paper will add further evidence to the literature to help patients make informed decisions in keeping with their preferences and values. A key conclusion from this study is the importance of shared decision making to ensure that patient values, as well as individual symptoms and situations, are considered. N-of-1 trials may be used to further explore optimal individualized treatment plans [49], as well as randomized-controlled trials to build the evidence base for musicians with PRMD in general. 

## Figures and Tables

**Table 1:** Patient characteristics at baseline.

	Non-Cannabis Users	New Cannabis Users	Long-Term Cannabis Users	*p* Value
*n*	42	61	101	
Age in years (mean, SD)	51, 16.5	47, 17.0	50, 13.7	0.297
Male sex (%)	29 (69)	44 (72)	78 (77)	0.551
Adverse childhood experiences (0–10)	2 ± 2.04	2 ± 2.03	2 ± 1.94	0.961
On other analgesia (Y)	10 (24%)	12 (20%)	25 (25%)	0.752

Data are presented as mean ± standard deviation.

**Table 2:** Patient questionnaire data at baseline.

	Non-Cannabis Users	New Cannabis Users	Long-Term Cannabis Users	*p* Value
MPIIQM40	14.63 ± 9.95	14.41 ± 8.56	12.25 ± 9.38	0.228
MPIIQM50	21.66 ± 14.44	23.18 ± 15.63	22.04 ± 13.97	0.847
MPIIQM	36.3 ± 22.12	37.59 ± 22.49	34.11 ± 20.05	0.582
Stress	13.35 ± 10.49	16.53 ± 12.60	16.75 ± 10.96	0.257
Anxiety	8.95 ± 7.87	9.40 ± 10.23	10.38 ± 9.68	0.673
Depression	11.95 ± 10.87	10.83 ± 11.06	13.29 ± 11.54	0.402
DASS-21	33.90 ± 25.64	36.92 ± 30.17	40.42 ± 29.07	0.439
PSQI	6.15 ± 3.60	6.79 ± 3.84	6.97 ± 3.89	0.506
Time between visits (days)	163.24 ± 85.57	168.66 ± 48.15	172.31 ± 58.16	0.726

Data are presented as mean ± standard deviation.

**Table 3:** Medical cannabis dosing for new users and long-term users.

	New Cannabis Users		Long-Term Cannabis Users		
	Baseline	Six Months	Baseline	Six Months	*p*-Value
CBD (mg)	0	24.87 ± 12.86	21.48 ± 12.50	23.39 ± 15.60	0.074
THC (mg)	0	2.11 ± 1.45	3.74 ± 4.22	4.41 ± 5.18	0.080

Data are presented as mean ± standard deviation.

**Table 4:** Scores at baseline and approx. 6 months.

	Non-Cannabis Users			New Cannabis Users			Long-Term Cannabis Users		
	Baseline	6 m	*p* Value	Baseline	6 m	*p* Value	Baseline	6 m	*p* Value
MPIIQM40	14.63 ± 9.95	13.81 ± 9.56	0.689	14.41 ± 8.56	11.80 ± 9.00	0.006	12.25 ± 9.38	12.92 ± 9.34	0.294
MPIIQM50	21.66 ± 14.44	17.67 ± 14.96	0.035	23.18 ± 15.63	17.28 ± 14.94	0.002	22.04 ± 13.97	18.22 ± 14.70	0.009
MPIIQM	36.3 ± 22.12	31.48 ± 23.27	0.082	37.59 ± 22.49	29.08 ± 22.15	0.001	34.11 ± 20.05	31.14 ± 22.42	0.123
Stress	13.35 ± 10.49	10.67 ± 9.67	0.043	16.53 ± 12.60	14.56 ± 12.20	0.174	16.75 ± 10.96	14.28 ± 10.96	0.010
Anxiety	8.95 ± 7.87	6.48 ± 6.94	0.027	9.40 ± 10.23	8.62 ± 9.07	0.534	10.38 ± 9.68	8.29 ± 8.14	0.004
Depression	11.95 ± 10.87	9.52 ± 9.72	0.086	10.83 ± 11.06	10.16 ± 11.65	0.653	13.29 ± 11.54	10.08 ± 11.19	0.002
DASS-21	33.90 ± 25.64	26.67 ± 22.61	0.022	36.92 ± 30.17	33.34 ± 30.49	0.365	40.42 ± 29.07	32.64 ± 26.90	0.001
PSQI	6.15 ± 3.60	6.55 ± 3.88	0.242	6.79 ± 3.84	6.79 ± 3.30	1.00	6.97 ± 3.89	7.31 ± 3.38	0.277

Data are presented as mean ± standard deviation.

**Table 5:** Change in patient questionnaire scores after approx. 6 months.

	Non-Cannabis Users	New Cannabis Users	Long-Term Cannabis Users	*p* Value
MPIIQM40	−0.48 ± 7.65	−2.61 ± 7.15	0.83 ± 0.79	0.023
MPIIQM50	−4.38 ± 13.00	−5.90 ± 14.21	−3.80 ± 14.33	0.651
MPIIQM total	−4.86 ± 17.68	−8.51 ± 19.32	−2.97 ± 19.20	0.199
Stress	−2.62 ± 8.11	−2.10 ± 11.90	−2.48 ± 9.50	0.960
Anxiety	−2.24 ± 6.34	−0.82 ± 10.24	−2.09 ± 7.08	0.562
Depression	−2.38 ± 8.77	−0.66 ± 11.33	−3.21 ± 10.16	0.310
DASS-21	−7.24 ± 19.71	−3.57 ± 30.60	−7.77 ± 23.48	0.573
PSQI	0.48 ± 2.60	0.00 ± 3.39	0.34 ± 3.10	0.706

Data are presented as mean ± standard deviation.

## Data Availability

Data are contained within the article.
